# Microwaves from Mobile Phones Inhibit 53BP1 Focus Formation in Human Stem Cells More Strongly Than in Differentiated Cells: Possible Mechanistic Link to Cancer Risk

**DOI:** 10.1289/ehp.0900781

**Published:** 2009-10-23

**Authors:** Eva Markovà, Lars O.G. Malmgren, Igor Y. Belyaev

**Affiliations:** 1 Department of Genetics, Microbiology and Toxicology, Stockholm University, Stockholm, Sweden; 2 Laboratory of Molecular Genetics, Cancer Research Institute, Bratislava, Slovak Republic; 3 MAX-lab, Lund University, Lund, Sweden; 4 Laboratory of Radiobiology, General Physics Institute, Russian Academy of Science, Moscow, Russia

**Keywords:** 53BP1 foci, DNA double-strand breaks, microwaves, mobile phones, stem cells

## Abstract

**Background:**

It is widely accepted that DNA double-strand breaks (DSBs) and their misrepair in stem cells are critical events in the multistage origination of various leukemias and tumors, including gliomas.

**Objectives:**

We studied whether microwaves from mobile telephones of the Global System for Mobile Communication (GSM) and the Universal Global Telecommunications System (UMTS) induce DSBs or affect DSB repair in stem cells.

**Methods:**

We analyzed tumor suppressor TP53 binding protein 1 (53BP1) foci that are typically formed at the sites of DSB location (referred to as DNA repair foci) by laser confocal microscopy.

**Results:**

Microwaves from mobile phones inhibited formation of 53BP1 foci in human primary fibroblasts and mesenchymal stem cells. These data parallel our previous findings for human lymphocytes. Importantly, the same GSM carrier frequency (915 MHz) and UMTS frequency band (1947.4 MHz) were effective for all cell types. Exposure at 905 MHz did not inhibit 53BP1 foci in differentiated cells, either fibroblasts or lymphocytes, whereas some effects were seen in stem cells at 905 MHz. Contrary to fibroblasts, stem cells did not adapt to chronic exposure during 2 weeks.

**Conclusions:**

The strongest microwave effects were always observed in stem cells. This result may suggest both significant misbalance in DSB repair and severe stress response. Our findings that stem cells are most sensitive to microwave exposure and react to more frequencies than do differentiated cells may be important for cancer risk assessment and indicate that stem cells are the most relevant cellular model for validating safe mobile communication signals.

The intensity levels of exposure to microwaves (MWs) from mobile telephones are lower than the International Commission on Non-ionizing Radiation Protection (ICNIRP) standards, which are based on thermal effects of acute MW exposures (ICNIRP 1998). However, effects of prolonged exposure to nonthermal (NT) MWs at intensities comparable with those of mobile phones have also been observed in many studies that indicate a relationship between NT MW exposure and permeability of the brain–blood barrier (Nittby et al. 2008), cerebral blood flow (Huber et al. 2005), stress response (Blank and Goodman 2004), and neuronal damage (Salford et al. 2003). The data obtained by the comet assay (Diem et al. 2005; Lai and Singh 1997) and the micronuclei assay (d’Ambrosio et al. 2002; Trosic et al. 2002; Zotti-Martelli et al. 2005) imply possible genotoxic effects of NT MWs, whereas other studies did not support this genotoxicity (Meltz 2003). Experimental data have indicated that the NT MW effects occur depending on several physical parameters, including carrier frequency, polarization, modulation, and intermittence (Belyaev 2005a). Differences in these physical parameters and biological variables, including genetic background and physiologic state, may explain various outcomes of studies with NT MWs (Belyaev 2005b; Huss et al. 2007).

A recent review of available epidemiologic studies concluded that the use of mobile phones for > 10 years is associated with increased risk of ipsilateral gliomas and acoustic neurinomas (Hardell et al. 2008). For a long time stem cells have been considered an important cellular target for origination of cancer—both tumors and leukemia (Feinberg et al. 2006; Soltysova et al. 2005). Gliomas are believed to originate from stem cells in the brain (Altaner 2008). DNA double-strand breaks (DSBs) and their misrepair are critical molecular events resulting in chromosomal aberrations, which have often been associated with origination of various leukemias and tumors, including gliomas (Fischer and Meese 2007). Only one study on possible MW-induced DSBs in stem cells is available (Nikolova et al. 2005). Surprisingly, the data obtained in that study by the neutral comet assay suggested that prolonged exposure time abolished the DSB formation observed at the shorter exposure time. Furthermore, the neutral comet assay has limited applicability to detect DSBs because similar increases in comet tails may be also caused by nongenotoxic effects that imply changes in chromatin conformation, such as relaxation of DNA loops (Belyaev et al. 1999).

Several proteins involved in DSB repair, such as phosphorylated histone 2A family member X (γ-H2AX) and tumor suppressor TP53 binding protein 1 (53BP1), have been shown to produce discrete foci that colocalize to DSBs, referred to as DNA repair foci (Kao et al. 2003; Sedelnikova et al. 2002). Analysis of DNA repair foci is currently accepted as the most sensitive and specific technique for measuring DSBs in untreated cells, as well as in cells exposed to cytotoxic agents (Bocker and Iliakis 2006; Bonner et al. 2008). By analysis of the DNA repair foci in normal human fibroblasts, we were able to detect DSBs induced by a very low dose of ionizing radiation, 1 cGy, which results in only 0.4 DSB/cell on average (Markovà et al. 2007). We have also used this technique to analyze 53BP1/γ-H2AX foci in human lymphocytes exposed to MWs from Global System for Mobile Communication (GSM)/Universal Global Telecommunications System (UMTS) phones (Belyaev et al. 2005, 2009; Markovà et al. 2005). We have found that MW exposure inhibited formation of endogenous 53BP1/γ-H2AX foci (Belyaev et al. 2005, 2009; Markovà et al. 2005). This inhibition might be caused by a decrease in accessibility of DSBs to proteins because of stress-induced chromatin condensation (Belyaev et al. 2009). Inability to form DNA repair foci has been correlated to radiosensitivity, genomic instability, and other repair defects (Bassing et al. 2002; Celeste et al. 2002; Kuhne et al. 2004; Olive and Banath 2004; Taneja et al. 2004). Inhibition of DSB repair may lead to chromosomal aberrations by either illegitimate recombination events (Bassing and Alt 2004) or reduced functionality of nonhomologous end-joining (Fischer and Meese 2007). Therefore, if similar effects on endogenous DNA repair foci are detected in stem cells, this might provide a direct mechanistic link to the epidemiologic data showing association of MW exposure with increased cancer risk.

Although γ-H2AX foci have been used to analyze endogenous and induced DSBs in most studies, recent data have indicated that γ-H2AX foci may also be produced by chromatin structure alternations and may not contain DSBs (Banath et al. 2004; Han et al. 2006; Suzuki et al. 2006; Yu et al. 2006). Accordingly, some γ-H2AX foci may not associate with DNA damage-response proteins such as 53BP1 (Belyaev et al. 2009; Markovà et al. 2005, 2007; McManus and Hendzel 2005). High expression of endogenous γ-H2AX in pluripotent mouse embryonic stem cells (~ 100 large γ-H2AX foci per cell) was not explained by DSBs, DNA degradation, or apoptosis, but it was attributed to the unusual organization of chromatin in mouse embryonic stem cells (Banath et al. 2009). The number of endogenous 53BP1 foci (< 3 foci/nucleus) appeared normal in mouse embryonic stem cells and is comparable with that found in other cell types (Banath et al. 2009). In contrast to γ-H2AX foci, which may be produced by the DSB-relevant and DSB-unrelated mechanisms, 53BP1 is relocalized to DSBs, along with other DNA damage-response proteins, such as phosphorylated ATM (ataxia telangiectasia mutated), Rad50, and MRE11 (meiotic recombination 11), and there is no indication that DSB-unrelated events would result in the formation of the 53BP1 foci (Medvedeva et al. 2007; Yoshikawa et al. 2009). Therefore, in this study we analyzed only 53BP1 foci as a more relevant marker for DSBs.

The differences in the DSB repair pathways between mouse and human stem cells have been described (Banuelos et al. 2008). In general, the comparisons of stem cells across species suggest that significant differences may be observed, so extrapolation from animal stem cell models to human health risk assessment should be done with care (Brons et al. 2007; Ginis et al. 2004). For the present study, we chose human adipose-tissue derived mesenchymal stem cells (MSCs). This cell type displays multipotency with the ability under the correct conditions to differentiate into lineages that cover a wide range of organs and tissues, such as bone, fat, cartilage, muscle, lung, skin, hepatocytes, and neurons (Bunnell et al. 2008; Porada et al. 2006; Sasaki et al. 2008). Of note, MSCs are at higher risk of malignant transformation than are embryonic stem cells (Soltysova et al. 2005).

In contrast to GSM exposure at the frequency of 915 MHz that consistently inhibited DNA repair foci in lymphocytes from 26 persons in total, GSM exposure at 905 MHz did not inhibit DNA repair focus formation, thereby providing evidence that MW effects depend on carrier frequency (Belyaev et al. 2005, 2009; Markovà et al. 2005). In previous studies we investigated MW effects on lymphocytes. However, it would be of interest to analyze the response of human stem cells, which are usually exposed to the mobile phone MWs along with differentiated human cells such as lymphocytes and fibroblasts. Therefore, in the present study we exposed human stem cells and primary human fibroblasts to GSM/UMTS MWs at the same frequencies as we used previously in experiments with human lymphocytes.

## Materials and Methods

### Cells

Human diploid VH-10 fibroblasts from the foreskin of a normal boy (a gift from A. Kolman, Department of Molecular Biology and Genome Research, Stockholm University) were maintained at 5% CO_2_ and 37°C in a humidified incubator as previously described (Markovà et al. 2007). Human MSCs separated from adipose tissue of two healthy persons (described previously by Kucerova et al. 2007), a gift from V. Altanerova (Cancer Research Institute), were cultivated in minimal essential medium (Alpha Medium, low glucose; Gibco Invitrogen, Carlsbad, CA, USA) supplemented with 10% MSC-stimulating supplement (human; StemCell Technologies, Grenoble, France) and 1% antibiotic/antimycotic mix (Gibco Invitrogen; concentration per milliliter of medium: penicillin, 100 U; streptomycin, 100 μg; Fungizone, 0.25 μg). Suspensions of cells 1 × 10^5^ (MSC) or 2 × 10^5^ (VH-10) in 3 mL of medium were seeded on cover slides in Petri dishes (35 × 10 mm; Sarstedt, Nümbrecht, Germany) and incubated at 37°C in 5% CO_2_ humidified atmosphere for 36–40 hr until reaching 80% confluence of cells.

### Cell exposure

Cells were exposed to GSM/UMTS MWs essentially as described previously (Belyaev et al. 2009; Sarimov et al. 2004). Briefly, exposures were performed using two specially designed installations, each based on a transverse electromagnetic line cell (TEM-cell) and a test mobile phone. The output of each phone was connected by the coaxial cable to the corresponding TEM-cell. Cells were exposed to either GSM (905 MHz or 915 MHz) or UMTS (1947.4 MHz, middle channel), with identical output power (0.25 W), at least three times for each exposure condition. All exposures were performed at 37°C in a 5% CO_2_ incubator using Petri dishes containing 3 mL medium per dish. The specific absorption rate (SAR) was 37 mW/kg for the 905/915 MHz frequency and 39 mW/kg for the 1947.4 MHz frequency. Taking into account all possible uncertainties, the SAR values at all locations within exposed samples were always well below thermal effects. Temperature was measured in the MW-exposed samples before, during, and after exposure with a precision of 0.1°C. No changes in temperature were induced in the samples during exposures.

In addition to MWs, mobile phones emit electromagnetic fields of extremely low frequency (ELF) that can also contribute to the exposure effects (Weisbrot et al. 2003). To avoid eventual effects of ELF exposure, the test mobile phones were situated 1 m from the CO_2_ incubator containing exposed samples. Accordingly, the ELF emission of our test mobile phones did not increase background ELF field, which did not exceed 200 nT (root mean square), as measured with a three-dimensional microteslameter (Field Dosimeter 3; Combinova, Bromma, Sweden) at the location of MW exposure.

We performed sham exposures in the same TEM-cells with MW power off. The order of MW- and sham-exposures was randomized among sessions. In each experiment, the sham exposures were performed in duplicate in the TEM-cell for GSM exposure and in the TEM-cell for UMTS exposure. No differences were observed between sham-exposed samples (sham – sham exposures). Therefore, we compared the MW effects with reference to combined sham-exposures. Heat treatment (41°C) was used as a positive control for stress response. As a positive control for genotoxic effect, the cells were irradiated with ^137^Cs γ-rays (3 Gy) using a Gammacell 1000 (Atomic Energy of Canada Limited, Ottawa, Canada) source at 10.6 Gy/min.

### Immunostaining and foci analysis

Immediately after exposure, the cells were placed on ice for 1 hr to prevent repair of eventual DSBs. The immunostaining was performed essentially as described previously (Markovà et al. 2005, 2007). The images were recorded from 5–10 fields of vision that were randomly selected from two slides on a Zeiss Axiovert 100M confocal laser scanning microscope using a plan-apochromat 63×/1.4 numerical aperture oil-immersion objective and LSM 510 software (LSM Image Browser 4.2.0.121; Carl Zeiss Microscopy, Jena, Germany). Through-focus maximum projection images were acquired from optical sections 1.00 μm apart and with a section thickness of 2.00 μm in the *z*-axis. Resolutions in the *x*- and *y*-axes were 0.20 μm. Eight optical sections were usually obtained for each field of vision, and the final image was obtained by projection of all sections onto one plane. For each independent exposure experiment and for each exposure condition (type of cell, type of exposure, exposure duration), we analyzed approximately 300 cells in double-blind fashion.

### Statistical analysis

We used Statistica 8.0 (StatSoft Inc., Tulsa, OK, USA) and SPSS Statistics 17.0 (SPSS Inc., Chicago, IL, USA) software for statistical analyses, according to the manufacturer’s instructions. Using analysis of variance (ANOVA) for several means, we set the statistical power to 0.80 based on estimates of sample variation and effect size obtained in our pilot experiments. The cell distributions of foci were analyzed using the Kolmogorov-Smirnov test. Most data did not fulfill the Poisson distribution. We analyzed either mean values from independent experiments or all raw data representing foci in each individual cell, using both nonparametric and parametric statistics. Bonferroni adjustment was used in multiple comparisons by ANOVA. In general, all methods provided similar results and conclusions. Results were considered significantly different at *p* < 0.05.

## Results

**Figure 1:**
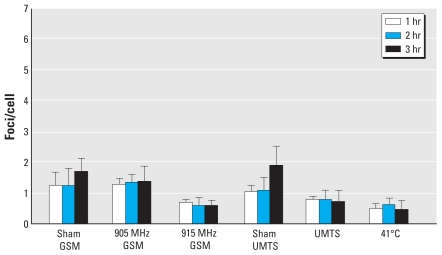
53BP1 foci in VH-10 fibroblasts after 1-, 2-, or 3-hr exposure to GSM MWs at 905 or 915 MHz, UMTS MWs at 1947.4 MHz, or heat shock at 41°C, as determined by immunostaining and confocal laser microscopy. Values shown are mean ± SD of cells from three to five experiments.

Both in fibroblasts and in MSCs, γ-irradiation (3 Gy) led to significant increases in 53BP1 foci caused by radiation-induced DSBs. In accordance with previously published data (Markovà et al. 2007), 26 foci/cell were found in fibroblasts 2 hr after irradiation, and a slightly higher level, 32 foci/cell, was detected in MSCs [see Supplemental Material, Figure 1 (doi:10.1289/ehp.0900781.S1 via dx.doi.org)]. Although we saw approximately one endogenous 53BP1 focus/cell in sham-exposed fibroblasts (see Supplemental Material, Figure 2), we observed a distinct MW-induced reduction in the level of these foci in response to 915 MHz (Figure 1). UMTS MWs also consistently reduced formation of endogenous 53BP1 foci in fibroblasts (Figure 1). Of note, the MW-induced reduction in 53BP1 foci was the same regardless of the duration of exposure within 1–3 hr, showing that saturation in the effects occurred after a 1-hr exposure (Figure 1). Analysis with the factorial ANOVA confirmed that the data did not depend on the exposure time. To verify the hypothesis that MW exposure for 1–3 hr affected formation of 53BP1 foci, we compared the effects using the Kruskal-Wallis ANOVA by ranks, the median test, and ANOVA. All tests showed that MWs affected formation of 53BP1 foci at highly significant levels (*p* < 0.001). Multiple comparisons showed significant effects of 915 MHz (*p* < 0.003) and UMTS (*p* < 0.01) at 1–3 hr exposure. On the other hand, exposure at 905 MHz did not affect fibroblasts. We observed a statistically significant difference between effects of 915 MHz and 905 MHz exposure (*p* < 0.01). These data parallel our findings for human lymphocytes (Belyaev et al. 2009) and suggest that both lymphocytes and fibroblasts respond to MWs at the same carrier frequencies, whereas other carrier frequencies do not affect these cells. Heat shock significantly inhibited formation of 53BP1 foci, similar to 915 MHz and UMTS MWs (*p* < 0.001). These data are in accordance with our previous findings for human lymphocytes (Belyaev et al. 2005, 2009; Markovà et al. 2005), suggesting that NT MW exposure at specific carrier frequencies induces stress responses similar to heat shock.

**Figure 2:**
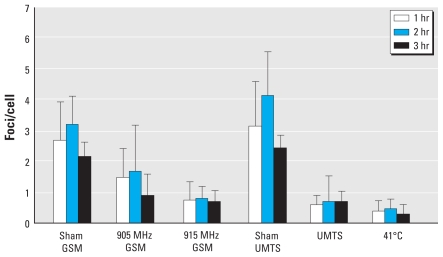
53BP1 foci in human MSCs after 1-, 2-, or 3-hr exposure to GSM MW at 905 or 915 MHz, UMTS MW at 1947.4 MHz, or heat shock at 41°C. Values shown are mean ± SD from three to five experiments.

The levels of endogenous 53BP1 foci in MSCs were approximately double those in fibroblasts [Figure 2; see also Supplemental Material, Figure 3 (doi:10.1289/ehp.0900781.S1)]. These data parallel the findings of others with mouse embryonic stem cells (Banath et al. 2009). Interestingly, we detected almost no foci in mitotic spreads of chromosomes of both MSCs and fibroblasts. The level of foci in mitotic cells was statistically significantly lower than in interphase cells (data not shown). These results are in line with previously published data indicating that many endogenous 53BP1 foci may not pass mitosis (Markovà et al. 2007).

**Figure 3:**
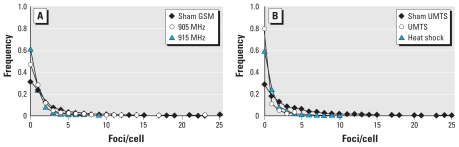
Distribution of 53BP1 foci among MSCs exposed to GSM (*A*) or UMTS (*B*) MWs. Distribution of cells according to number of foci per cell is shown as normalized frequency of cells versus the number of foci per cell. Heat shock (41°C) served as the positive control for MW exposure.

Similar to our findings for fibroblasts, we observed a distinct MW-induced reduction in the level of endogenous 53BP1 foci in MSCs exposed to 915 MHz and UMTS MW (Figure 2). However, these inhibitory effects of MW exposures were about 2-fold more pronounced in MSCs than in fibroblasts (Figures 1 and 2). As shown in Figure 2, prolongation of exposure did not result in increased inhibition, providing evidence that effects of MW exposure saturated at 1 hr of exposure. Analysis with factorial ANOVA confirmed that the data did not depend on exposure time. The Kruskal-Wallis ANOVA by ranks, the median test, and ANOVA showed that MWs affected formation of 53BP1 foci at very highly significant levels (*p* < 0.0001). The effects of exposure to 915 MHz and UMTS for 1–3 hr were highly significant (*p* < 0.0005). In contrast to fibroblasts, approximately 5% of MSCs had multiple foci [> 10 foci/cell; see Supplemental Material, Figure 4 (doi:10.1289/ehp.0900781.S1)]. The origin of these foci is unknown, but they were completely inhibited by MW exposure (Figure 3). Heat shock at 41°C also inhibited formation of 53BP1 foci in MSCs (Figure 2), although this inhibition was stronger than in the heat-shocked fibroblasts (Figure 1). Altogether, the data provide evidence that exposure to 915 MHz or UMTS MWs, as well as heat shock, results in stronger stress response in MSCs than in fibroblasts. Although we observed some reduction in formation of foci after exposure of MSCs to GSM MW at 905 MHz (Figures 2 and 3), this effect was not statistically significant. The effects of 905 MHz and 915 MHz were also not statistically different. These findings indicate that MWs may affect MSCs at more carrier frequencies compared with differentiated cells.

**Figure 4:**
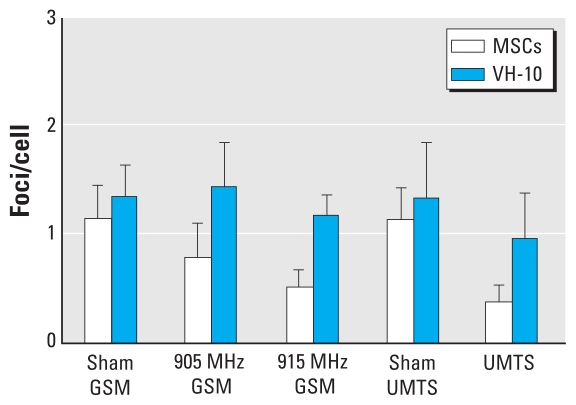
53BP1 foci in VH-10 fibroblasts and MSCs after chronic exposure during 10 days (5 days/week, 1 hr/day) to GSM MW at 905 MHz or 915 MHz, and UMTS MW at 1947.4 MHz. Values shown are mean ± SD from three experiments.

We further tested whether MSCs and fibroblasts can adapt to MW effects during chronic exposure by exposing the cells for 2 weeks (5 days/week, 1 hr/day). Interestingly, MSCs with multiple foci almost disappeared during 2 weeks of cultivation of untreated cells. Thus, the levels of endogenous foci did not differ between MSCs and fibroblasts (Figure 4). Fibroblasts almost completely adapted to the chronic MW exposure (Figure 4); however, we saw no such adaptation in MSCs. All statistical tests we used showed that chronic MW exposure affected formation of 53BP1 foci in MSCs (*p* < 0.05, multiple comparisons by Kruskal-Wallis ANOVA by ranks and median tests; *p* < 0.001, ANOVA). Inhibitory effects of MW exposures at the 915 MHz GSM and 1947.4 MHz UMTS were statistically significant during the 2 week exposure of MSCs (*p* < 0.05, Kruskal-Wallis ANOVA; *p* < 0.005, ANOVA). Comparison of arrays containing data from each individual cell confirmed that chronic MW exposure resulted in significant effects in MSCs [see Supplemental Material, Statistics (doi:10.1289/ehp.0900781.S1)]. In addition, these comparisons revealed the effect of 905 MHz GSM exposure and showed that UMTS exposure affected MSCs more strongly than did the GSM exposures.

## Discussion

We report here for the first time that exposure of human MSCs and human primary fibroblasts to MWs from GSM/UMTS mobile phones inhibits formation of endogenous 53BP1 foci. Similar although not the same inhibitory effects of MWs from GSM/UMTS mobile phones have previously been found in primary human lymphocytes (Belyaev et al. 2009). We used these cell types for two main reasons. First, the emerging data show that effects of low-intensity MWs are cell-type dependent (Sanchez et al. 2006; Schwarz et al. 2008). In particular, immortalized and primary cells may respond differently to MWs. Therefore, the data obtained with human primary cells would be of utmost relevance for assessing possible health risks of MW exposure from mobile phones. Second, it now appears that most, if not all, adult tissues and organs, including blood, skin, and brain, contain stem cells (Metcalfe and Ferguson 2008). Therefore, stem cells, like blood cells and fibroblasts, are always subjected to exposure from mobile phones.

Our data indicate that fibroblasts are more resistant to MWs than are MSCs (present study) and human peripheral blood lymphocytes (Belyaev et al. 2009). Moreover, we show here that fibroblasts are able to adapt to MWs during chronic exposure. These results are consistent with the suggestion that adaptive cell behavior in response to MW exposure is unlikely to have adverse effects at the skin level (Sanchez et al. 2006). However, we saw no adaptation in MSCs. Thus, although our findings with chronic exposure of fibroblasts may suggest no health risks at the skin level, high sensitivity of stem cells may imply such risks.

No heat was induced in the samples exposed to MW. The SAR values at different locations of the exposed samples were always well below thermal effects. Therefore, the MW effects could not be attributed to heating, although we observed a similar response after both MW exposure and heat shock. This similarity indicates that MW exposure at 915 MHz/1947.4 MHz is a stress factor for fibroblasts and especially for human stem cells, where we saw stronger effects.

Modifications of 53BP1, such as phosphorylation, are needed for repair of DSBs (Ward et al. 2006). Thus, our finding on the inhibition of DNA repair foci can be accounted for by inhibition of phosphorylation of 53BP1 protein. Experimental evidence for such a mechanism has recently been reported (Leszczynski et al. 2002). Alternatively, MW exposure can result in chromatin condensation that prevents DSBs from accessing DNA repair proteins (Belyaev et al. 2005; Markovà et al. 2005; Sarimov et al. 2004). Regardless of the molecular mechanism, inhibition of DSB repair in stem cells may result in chromosomal aberrations by either illegitimate recombination events (Bassing and Alt 2004) or reduced functionality of nonhomologous end-joining (Fischer and Meese 2007).

We have found that the constitutive level of 53BP1 foci in human MSCs is significantly higher than in differentiated primary human cells such as fibroblasts (present study) and lymphocytes (Belyaev et al. 2009). Importantly, we did not observe adaptation to NT GSM/UMTS MW chronic exposure in stem cells. Altogether, our findings show that human stem cells are more sensitive than differentiated primary cells to MW exposure from mobile phones. Thus, inhibition of 53BP1 foci in stem cells may account for higher risks in these cells than in differentiated cells with lower constitutive 53BP1 levels.

In the present study we found that the inhibitory effect of MWs on the 53BP1 foci leveled off at 1 hr of exposure, and we observed no further increase in effects both in MSCs and fibroblasts after prolonging exposure to 3 hr. These data are in agreement with previous results that MW effects were the same at 1 hr and 2 hr exposures in human peripheral blood lymphocytes (Belyaev et al. 2005; Markovà et al. 2005). Preliminary data indicate that saturation in the MW effect is observed at an even shorter exposure time (30 min), whereas almost linear dependence on exposure time is present within shorter exposure times (Belyaev IY, Markovà E, unpublished data).

Both the 1947.4 MHz UMTS frequency band and the 915 MHz GSM signal affected all tested human cell types: stem cells and fibroblasts (present study) and lymphocytes (Belyaev et al. 2009). On the other hand, MW exposure at another GSM frequency (905 MHz) did not result in statistically significant effects in lymphocytes or fibroblasts. Thus, GSM MW exposure may either inhibit or not inhibit DNA repair foci depending on carrier frequency. Neither SAR nor the SAR measurement uncertainty depended on carrier frequency in the range of 905–915 MHz. Therefore, the difference in the effects at 905 and 915 MHz could not be attributed to the differences in the MW absorption. The “frequency” and “intensity” windows have often been reported for NT MW effects (for review, see Belyaev 2005b; Blackman 1992; Grundler 1992). Correspondingly, there may be “effective” and “ineffective” carrier GSM frequencies that either affect human cells or induce no effect. Several physical mechanisms have been suggested to account for the frequency-dependent effects of NT MW (Belyaev et al. 1996; Binhi 2002; Kaiser 1995; Matronchik and Belyaev 2008). Our previous findings indicated that the intensity windows may not coincide for various carrier frequencies (Belyaev et al. 1996; Shcheglov et al. 1997). Correspondingly, the SAR value of 39 mW/kg used here may be optimal for the effects at 915 MHz but not for those at 905 MHz. Alternatively, but less likely, it is possible that the cells have molecular components that have different electrical properties, thus altering the effective intensity (Joines and Blackman 1980). In either case, future testing of the cell response as a function of exposure intensities at 905 and 915 MHz should help to resolve this issue. Regardless of physical mechanism, our findings suggest that specific carrier frequencies and bands that do not induce adverse effects can be validated in laboratory studies with primary human cells as the prerequisite for the development of safe wireless technologies.

Although we saw no statistically significant effects in stem cells exposed to 905 MHz, by comparing mean values, we observed a trend to inhibition of the DNA repair foci in these cells both under acute and chronic exposures (Figures 2–4). Moreover, the MW effects at 905 and 915 MHz were not statistically significantly different in stem cells, and analysis of the individual cell arrays revealed effects of exposure to 905 MHz. These findings indicate that stem cells may react to more frequencies than do differentiated primary human cells. Higher biological significance of MW effects in stem cells and apparently wider range of effective frequencies suggest that stem cells are the most relevant cellular model for assessment of health risks from mobile communication.

Endogenous 53BP1 foci are typically considered sensitive markers for endogenous DSBs, resulting in intrinsic genomic instability (Adams and Carpenter 2006; Banath et al. 2009; Sedelnikova et al. 2008). However, 53BP1 foci represent only indirect DSB measurements. There is no direct evidence that 53BP1 plays a role in the repair of endogenous DSBs. If irrelevance of the endogenous 53BP1 foci to DSBs is proven, the MW effects described here should be interpreted solely as a manifestation of stress response. This alternative interpretation is supported by the data that MW exposure inhibits 53BP1 foci similar to heating of cells (Figures 1–3). Stress response has previously been suggested as a criterion for adverse effects of electromagnetic fields (Blank and Goodman 2004). In fact, the currently accepted safety standards assume that MW exposure is harmful only if its effects are similar to those of heating (ICNIRP 1998). Stress may be especially important for stem cells because it is believed to be an important factor in the multistage origination of cancer from human stem cells (Feinberg et al. 2006; Tez 2008). Both interpretations of the data—either disruption of the balance between cellular repair systems and DNA damage or stress response—are not mutually exclusive, and both may provide a mechanistic link to the epidemiologic data showing association of prolonged MW exposure with brain cancer risk (Hardell et al. 2008). It should also be mentioned that stress can reduce neurogenesis (Sohur et al. 2006).

The best indications of the role of stem cells in cancer arise from hematologic disorders such as leukemia. In several epidemiologic studies, ELF exposure has been associated with increased childhood leukemia (Kabuto et al. 2006). On the other hand, no association of ELF exposure with leukemia has been found in adults. This discrepancy has not yet been clarified at the mechanistic level, although ELF has been classified as a possible carcinogen based on these studies (International Agency for Research on Cancer 2002). In a recent study Yang et al. (2008) suggested a possible association between electric transformers and power lines and the *XRCC1* Ex9+16A allele in patients with childhood acute leukemia. ELF exposure has often been reported to result in biological effects similar to those caused by exposures to NT MWs (Adey 1981; Blackman 1992), and ELF and MW exposures similarly inhibited formation of DNA repair foci in human lymphocytes (Belyaev et al. 2005). Stem cells are more active in children than in adults (Williams et al. 2006). This increased activity of stem cells may clarify the differences between results obtained in ELF–leukemia studies with children and adults and may call for studies on possible cancer risks of MW exposure of children.

## Conclusion

We have demonstrated that GSM/UMTS MWs from mobile phones inhibit formation of endogenous 53BP1 foci in human primary fibroblasts and MSCs. In contrast to fibroblasts, MSCs did not adapt to MWs during chronic exposure. Together, our results indicate that stem cells are more sensitive to MW exposure than are differentiated human primary cells, lymphocytes, and fibroblasts, whereas fibroblasts are least sensitive. Inhibitory effects of MW exposure on DSB repair in stem cells may result in formation of chromosomal aberrations and therefore origination of cancer. Alternatively, MW exposures may induce a stress response. Both possible interpretations provide a mechanistic link to increased cancer risk. Our finding that MSCs may react to more carrier frequencies than differentiated cells may indicate that stem cells are the most relevant cellular model for validating safe mobile communication signals. Because almost all organs and tissues possess stem cells and because stem cells are more active in children, the possible relationship of chronic MW exposure and various types of tumors and leukemia—especially in children—should be investigated.

## References

[b1-ehp-118-394] Adams MM, Carpenter PB (2006). Tying the loose ends together in DNA double strand break repair with 53BP1. Cell Div.

[b2-ehp-118-394] Adey WR (1981). Tissue interactions with nonionizing electromagnetic fields. Physiol Rev.

[b3-ehp-118-394] Altaner C (2008). Glioblastoma and stem cells. Neoplasma.

[b4-ehp-118-394] Banath JP, Banuelos CA, Klokov D, MacPhail SM, Lansdorp PM, Olive PL (2009). Explanation for excessive DNA single-strand breaks and endogenous repair foci in pluripotent mouse embryonic stem cells. Exp Cell Res.

[b5-ehp-118-394] Banath JP, Macphail SH, Olive PL (2004). Radiation sensitivity, H2AX phosphorylation, and kinetics of repair of DNA strand breaks in irradiated cervical cancer cell lines. Cancer Res.

[b6-ehp-118-394] Banuelos CA, Banath JP, MacPhail SH, Zhao J, Eaves CA, O’Connor MD (2008). Mouse but not human embryonic stem cells are deficient in rejoining of ionizing radiation-induced DNA double-strand breaks. DNA Repair (Amst).

[b7-ehp-118-394] Bassing CH, Alt FW (2004). H2AX may function as an anchor to hold broken chromosomal DNA ends in close proximity. Cell Cycle.

[b8-ehp-118-394] Bassing CH, Chua KF, Sekiguchi J, Suh H, Whitlow SR, Fleming JC (2002). Increased ionizing radiation sensitivity and genomic instability in the absence of histone H2AX. Proc Natl Acad Sci USA.

[b9-ehp-118-394] Belyaev I (2005a). Nonthermal biological effects of microwaves: current knowledge, further perspective, and urgent needs. Electromagn Biol Med.

[b10-ehp-118-394] Belyaev I (2005b). Non-thermal biological effects of microwaves. Microwave Rev.

[b11-ehp-118-394] Belyaev IY, Eriksson S, Nygren J, Torudd J, Harms-Ringdahl M (1999). Effects of ethidium bromide on DNA loop organisation in human lymphocytes measured by anomalous viscosity time dependence and single cell gel electrophoresis. Biochim Biophys Acta.

[b12-ehp-118-394] Belyaev IY, Hillert L, Protopopova M, Tamm C, Malmgren LO, Persson BRR (2005). 915 MHz microwaves and 50 Hz magnetic field affect chromatin conformation and 53BP1 foci in human lymphocytes from hypersensitive and healthy persons. Bioelectromagnetics.

[b13-ehp-118-394] Belyaev IY, Markovà E, Hillert L, Malmgren LO, Persson BR (2009). Microwaves from UMTS/GSM mobile phones induce long-lasting inhibition of 53BP1/gamma-H2AX DNA repair foci in human lymphocytes. Bioelectromagnetics.

[b14-ehp-118-394] Belyaev IY, Shcheglov VS, Alipov YD, Polunin VA (1996). Resonance effect of millimeter waves in the power range from 10^−19^ to 3 x 10^−3^ W/cm^2^ on *Escherichia coli* cells at different concentrations. Bioelectromagnetics.

[b15-ehp-118-394] Binhi VN (2002). Magnetobiology: Underlying Physical Problems.

[b16-ehp-118-394] Blackman CF, Norden B, Ramel C (1992). Calcium release from nervous tissue: experimental results and possible mechanisms. Interaction Mechanisms of Low-Level Electromagnetic Fields in Living Systems.

[b17-ehp-118-394] Blank M, Goodman R (2004). Comment: a biological guide for electromagnetic safety: the stress response. Bioelectromagnetics.

[b18-ehp-118-394] Bocker W, Iliakis G (2006). Computational methods for analysis of foci: validation for radiation-induced gamma-H2AX foci in human cells. Radiat Res.

[b19-ehp-118-394] Bonner WM, Redon CE, Dickey JS, Nakamura AJ, Sedelnikova OA, Solier S (2008). γH2AX and cancer. Nat Rev.

[b20-ehp-118-394] Brons IG, Smithers LE, Trotter MW, Rugg-Gunn P, Sun B, Chuva de Sousa Lopes SM (2007). Derivation of pluripotent epiblast stem cells from mammalian embryos. Nature.

[b21-ehp-118-394] Bunnell BA, Estes BT, Guilak F, Gimble JM (2008). Differentiation of adipose stem cells. Methods Mol Biol.

[b22-ehp-118-394] Celeste A, Petersen S, Romanienko PJ, Fernandez-Capetillo O, Chen HT, Sedelnikova OA (2002). Genomic instability in mice lacking histone H2AX. Science.

[b23-ehp-118-394] d’Ambrosio G, Massa R, Scarfi MR, Zeni O (2002). Cytogenetic damage in human lymphocytes following GMSK phase modulated microwave exposure. Bioelectromagnetics.

[b24-ehp-118-394] Diem E, Schwarz C, Adlkofer F, Jahn O, Rudiger H (2005). Non-thermal DNA breakage by mobile-phone radiation (1800 MHz) in human fibroblasts and in transformed GFSH-R17 rat granulosa cells in vitro. Mutat Res.

[b25-ehp-118-394] Feinberg AP, Ohlsson R, Henikoff S (2006). The epigenetic progenitor origin of human cancer. Nat Rev Genet.

[b26-ehp-118-394] Fischer U, Meese E (2007). Glioblastoma multiforme: the role of DSB repair between genotype and phenotype. Oncogene.

[b27-ehp-118-394] Ginis I, Luo Y, Miura T, Thies S, Brandenberger R, Gerecht-Nir S (2004). Differences between human and mouse embryonic stem cells. Dev Biol.

[b28-ehp-118-394] Grundler W (1992). Intensity- and frequency-dependent effects of microwaves on cell growth rates. Bioelectrochem Bioenerg.

[b29-ehp-118-394] Han J, Hendzel MJ, Allalunis-Turner J (2006). Quantitative analysis reveals asynchronous and more than DSB-associated histone H2AX phosphorylation after exposure to ionizing radiation. Radiat Res.

[b30-ehp-118-394] Hardell L, Carlberg M, Soderqvist F, Hansson Mild K (2008). Meta-analysis of long-term mobile phone use and the association with brain tumours. Int J Oncol.

[b31-ehp-118-394] Huber R, Treyer V, Schuderer J, Berthold T, Buck A, Kuster N (2005). Exposure to pulse-modulated radio frequency electromagnetic fields affects regional cerebral blood flow. Eur J Neurosci.

[b32-ehp-118-394] Huss A, Egger M, Hug K, Huwiler-Muntener K, Roosli M (2007). Source of funding and results of studies of health effects of mobile phone use: systematic review of experimental studies. Environ Health Perspect.

[b33-ehp-118-394] ICNIRP (International Commission on Non-ionizing Radiation Protection) (1998). Guidelines for limiting exposure to time-varying electric, magnetic, and electromagnetic fields (up to 300 GHz). Health Physics.

[b34-ehp-118-394] International Agency for Research on Cancer (2002). Non-ionizing Radiation, Part I: Static and Extremely Low Frequency (ELF) Electric and Magnetic Fields. IARC Monogr Eval Carcinog Risk Hum.

[b35-ehp-118-394] Joines WT, Blackman CF (1980). Power density, field intensity, and carrier frequency determinants of RF-energy-induced calcium-ion efflux from brain tissue. Bioelectromagnetics.

[b36-ehp-118-394] Kabuto M, Nitta H, Yamamoto S, Yamaguchi N, Akiba S, Honda Y (2006). Childhood leukemia and magnetic fields in Japan: a case-control study of childhood leukemia and residential power-frequency magnetic fields in Japan. Int J Cancer.

[b37-ehp-118-394] Kaiser F (1995). Coherent oscillations—their role in the interaction of weak ELM-fields with cellular systems. Neural Netw World.

[b38-ehp-118-394] Kao GD, McKenna WG, Guenther MG, Muschel RJ, Lazar MA, Yen TJ (2003). Histone deacetylase 4 interacts with 53BP1 to mediate the DNA damage response. J Cell Biol.

[b39-ehp-118-394] Kucerova L, Altanerova V, Matuskova M, Tyciakova S, Altaner C (2007). Adipose tissue-derived human mesenchymal stem cells mediated prodrug cancer gene therapy. Cancer Res.

[b40-ehp-118-394] Kuhne M, Riballo E, Rief N, Rothkamm K, Jeggo PA, Lobrich M (2004). A double-strand break repair defect in ATM-deficient cells contributes to radiosensitivity. Cancer Res.

[b41-ehp-118-394] Lai H, Singh NP (1997). Melatonin and a spin-trap compound block radiofrequency electromagnetic radiation-induced DNA strand breaks in rat brain cells. Bioelectromagnetics.

[b42-ehp-118-394] Leszczynski D, Joenvaara S, Reivinen J, Kuokka R (2002). Non-thermal activation of the hsp27/p38MAPK stress pathway by mobile phone radiation in human endothelial cells: molecular mechanism for cancer- and blood-brain barrier-related effects. Differentiation.

[b43-ehp-118-394] Markovà E, Hillert L, Malmgren L, Persson BRR, Belyaev IY (2005). Microwaves from GSM mobile telephones affect 53BP1 and γ-H2AX foci in human lymphocytes from hypersensitive and healthy persons. Environ Health Perspect.

[b44-ehp-118-394] Markovà E, Schultz N, Belyaev IY (2007). Kinetics and dose-response of residual 53BP1/gamma-H2AX foci: co-localization, relationship with DSB repair and clonogenic survival. Int J Radiat Biol.

[b45-ehp-118-394] Matronchik AY, Belyaev IY (2008). Mechanism for combined action of microwaves and static magnetic field: slow non uniform rotation of charged nucleoid. Electromagn Biol Med.

[b46-ehp-118-394] McManus KJ, Hendzel MJ (2005). ATM-dependent DNA damage-independent mitotic phosphorylation of H2AX in normally growing mammalian cells. Mol Biol Cell.

[b47-ehp-118-394] Medvedeva NG, Panyutin IV, Panyutin IG, Neumann RD (2007). Phosphorylation of histone H2AX in radiation-induced micronuclei. Radiat Res.

[b48-ehp-118-394] Meltz ML (2003). Radiofrequency exposure and mammalian cell toxicity, genotoxicity, and transformation. Bioelectromagnetics.

[b49-ehp-118-394] Metcalfe AD, Ferguson MW (2008). Skin stem and progenitor cells: using regeneration as a tissue-engineering strategy. Cell Mol Life Sci.

[b50-ehp-118-394] Nikolova T, Czyz J, Rolletschek A, Blyszczuk P, Fuchs J, Jovtchev G (2005). Electromagnetic fields affect transcript levels of apoptosis-related genes in embryonic stem cell-derived neural progenitor cells. FASEB J.

[b51-ehp-118-394] Nittby H, Grafstrom G, Eberhardt JL, Malmgren L, Brun A, Persson BR (2008). Radiofrequency and extremely low-frequency electromagnetic field effects on the blood-brain barrier. Electromagn Biol Med.

[b52-ehp-118-394] Olive PL, Banath JP (2004). Phosphorylation of histone H2AX as a measure of radiosensitivity. Int J Radiat Oncol Biol Phys.

[b53-ehp-118-394] Porada CD, Zanjani ED, Almeida-Porad G (2006). Adult mesenchymal stem cells: a pluripotent population with multiple applications. Curr Stem Cell Res Ther.

[b54-ehp-118-394] Salford LG, Brun AE, Eberhardt JL, Malmgren L, Persson BRR (2003). Nerve cell damage in mammalian brain after exposure to microwaves from GSM mobile phones. Environ Health Perspect.

[b55-ehp-118-394] Sanchez S, Milochau A, Ruffie G, Poulletier de Gannes F, Lagroye I, Haro E (2006). Human skin cell stress response to GSM-900 mobile phone signals. In vitro *s*tudy on isolated primary cells and reconstructed epidermis. FEBS J.

[b56-ehp-118-394] Sarimov R, Malmgren LOG, Markovà E, Persson BRR, Belyaev IY (2004). Non-thermal GSM microwaves affect chromatin conformation in human lymphocytes similar to heat shock. IEEE Trans Plasma Sci.

[b57-ehp-118-394] Sasaki M, Abe R, Fujita Y, Ando S, Inokuma D, Shimizu H (2008). Mesenchymal stem cells are recruited into wounded skin and contribute to wound repair by transdifferentiation into multiple skin cell type. J Immunol.

[b58-ehp-118-394] Schwarz C, Kratochvil E, Pilger A, Kuster N, Adlkofer F, Rudiger HW (2008). Radiofrequency electromagnetic fields (UMTS, 1,950 MHz) induce genotoxic effects in vitro in human fibroblasts but not in lymphocytes. Int Arch Occup Environ Health.

[b59-ehp-118-394] Sedelnikova OA, Horikawa I, Redon C, Nakamura A, Zimonjic DB, Popescu NC (2008). Delayed kinetics of DNA double-strand break processing in normal and pathological aging. Aging Cell.

[b60-ehp-118-394] Sedelnikova OA, Rogakou EP, Panyutin IG, Bonner WM (2002). Quantitative detection of (125)IdU-induced DNA double-strand breaks with gamma-H2AX antibody. Radiat Res.

[b61-ehp-118-394] Shcheglov VS, Belyaev IY, Ushakov VL, Alipov YD (1997). Power-dependent rearrangement in the spectrum of resonance effect of millimeter waves on the genome conformational state of *E. coli* cells. Electro- Magnetobiol.

[b62-ehp-118-394] Sohur US, Emsley JG, Mitchell BD, Macklis JD (2006). Adult neurogenesis and cellular brain repair with neural progenitors, precursors and stem cells. Philos Trans R Soc Lond B Biol Sci.

[b63-ehp-118-394] Soltysova A, Altanerova V, Altaner C (2005). Cancer stem cells. Neoplasma.

[b64-ehp-118-394] Suzuki M, Suzuki K, Kodama S, Watanabe M (2006). Phosphorylated histone H2AX foci persist on rejoined mitotic chromosomes in normal human diploid cells exposed to ionizing radiation. Radiat Res.

[b65-ehp-118-394] Taneja N, Davis M, Choy JS, Beckett MA, Singh R, Kron SJ (2004). Histone H2AX phosphorylation as a predictor of radiosensitivity and target for radiotherapy. J Biol Chem.

[b66-ehp-118-394] Tez M (2008). Cancer is an adaptation mechanism of the aged stem cell against stress. Rejuvenation Res.

[b67-ehp-118-394] Trosic I, Busljeta I, Kasuba V, Rozgaj R (2002). Micronucleus induction after whole-body microwave irradiation of rats. Mutat Res.

[b68-ehp-118-394] Ward I, Kim JE, Minn K, Chini CC, Mer G, Chen J (2006). The tandem BRCT domain of 53BP1 is not required for its repair function. J Biol Chem.

[b69-ehp-118-394] Weisbrot D, Lin H, Ye L, Blank M, Goodman R (2003). Effects of mobile phone radiation on reproduction and development in *Drosophila melanogaster*. J Cell Biochem.

[b70-ehp-118-394] Williams DA, Xu H, Cancelas JA (2006). Children are not little adults: just ask their hematopoietic stem cells. J Clin Invest.

[b71-ehp-118-394] Yang Y, Jin X, Yan C, Tian Y, Tang J, Shen X (2008). Case-only study of interactions between DNA repair genes (hMLH1, APEX1, MGMT, XRCC1 and XPD) and low-frequency electromagnetic fields in childhood acute leukemia. Leuk Lymphoma.

[b72-ehp-118-394] Yoshikawa T, Kashino G, Ono K, Watanabe M (2009). Phosphorylated H2AX foci in tumor cells have no correlation with their radiation sensitivities. J Radiat Res (Tokyo).

[b73-ehp-118-394] Yu T, MacPhail SH, Banath JP, Klokov D, Olive PL (2006). Endogenous expression of phosphorylated histone H2AX in tumors in relation to DNA double-strand breaks and genomic instability. DNA Repair (Amst).

[b74-ehp-118-394] Zotti-Martelli L, Peccatori M, Maggini V, Ballardin M, Barale R (2005). Individual responsiveness to induction of micronuclei in human lymphocytes after exposure *in vitro* to 1800-MHz microwave radiation. Mutat Res.

